# Is level of neighbourhood green space associated with physical activity in green space?

**DOI:** 10.1186/1479-5868-10-127

**Published:** 2013-11-13

**Authors:** Katherine Ord, Richard Mitchell, Jamie Pearce

**Affiliations:** 1 Institute of Health and Wellbeing, University of Glasgow, 1 Lilybank Gardens, Glasgow G128RZ, UK; 2 School of GeoSciences, University of Edinburgh, Drummond Street, Edinburgh EHE9XP, UK

**Keywords:** Green space, Physical activity, Health inequalities

## Abstract

**Background:**

There is accumulating evidence that greater availability of green space in a neighbourhood is associated with health benefits for the local population. One mechanism proposed for this association is that green space provides a venue for, and therefore encourages, physical activity. It has also been suggested that socio-economic health inequalities may be narrower in greener areas because of the equalised opportunity for physical activity green spaces provide. However, research exploring associations between the availability of green space and physical activity has produced mixed results. Limits to the assessment of the type and amount of physical activity which occurs specifically in green space may account for these mixed findings. This observational study was therefore concerned with the extent to which green space is a venue for physical activity and whether this could account for narrower socio-economic health inequalities in greener neighbourhoods.

**Method:**

Secondary analysis of cross sectional data on 3679 adults (16+) living in urban areas across Scotland matched with a neighbourhood level measure of green space availability. Associations between green space availability and both total physical activity, and activity specifically within green space, were explored using logistic regression models. Interactions between socio-economic position and physical activity were assessed. All models adjusted for age, sex and household income.

**Results:**

The availability of green space in a neighbourhood was not associated with total physical activity or that specifically in green space. There was no evidence that income-related inequalities in physical activity within green space were narrower in greener areas of Scotland.

**Conclusion:**

Physical activity may not be the main mechanism explaining the association between green space and health in Scotland. The direct effect of perceiving a natural environment on physiological and psychological health may offer an alternative explanation.

## Background

There is accumulating evidence that greater availability of green space in an urban neighbourhood is associated with health benefits for the local population [1-7]. Three plausible mechanisms have been proposed to explain why green space may exert a beneficial effect on health. First, contact with natural environments can promote restoration from stress and mental fatigue [8-10]. Second, green spaces may influence health by facilitating social interaction within a community [11,12]. Third, and the focus of this study, green space may encourage people to engage in physical activity by, for example, providing increased opportunities for walking and cycling [13,14].

However, research examining physical activity as a mechanism explaining the relationship between green space and health has produced mixed results. A recent systematic review identified 50 studies which explored whether the availability of green space affects physical activity levels of the local population [15]. Of these studies, only 40% reported a positive association. These mixed results were found at both a regional and national level. The review identified considerable variation in how access to green space has been measured. Some studies used distance to the nearest green space. Coombes et al. [16], for example, found that residents of Bristol, England who lived closer to green space were more likely to meet the recommended physical activity guidelines than those living further away. Hillsdon et al. [17], however, found no relationship between road distance to green space and recreational physical activity among adults in Norwich, England. Other studies have used the percentage of land area in a respondent’s neighbourhood that can be classified as green space. Mytton et al. [18], for example, found that residents of neighbourhoods with greater proportions of green space were more likely to meet recommended physical activity guidelines. In contrast, in the Netherlands Maas et al. [19] found no association between the percentage of green space in the neighbourhood and meeting the recommended physical activity guidelines.

The literature is also diverse in study design, setting and measurement of physical activity. This variation may explain the varied and consequently inconclusive results [14,15]. One of the least discussed, but perhaps most important, weaknesses in the literature is the assessment and understanding of the type and amount of physical activity which occurs specifically *in green space*. Studies, at a national level, often measure physical activity behaviour by using nationally relevant recommended physical activity guidelines. In the UK, for example, the recommendation is to accumulate 150 minutes of moderate intensity physical activity, in bouts of 10 minutes or more, throughout the week [14,16,17,20]. Survey data are often used to assess whether an individual meets the target, or how many minutes of activity have been accumulated towards it. This measurement typically captures physical activity across four domains: domestic, transport, recreational and occupational but only two (transport and recreational) are likely to include physical activity undertaken in green space. If we observe that a population living in a greener area achieves on average higher levels of physical activity on this kind of measure, we cannot assume that green space is implicated. This limitation, and the potential for miss-interpretation, was recently explored by Mytton et al. [18]. They found that neighbourhood level quantities of green space were positively associated with likelihood of meeting the recommended physical activity guidelines. Their study was limited by the fact that they did not know the types of environments in which their respondents were physically active, but they did know the types of physical activity the respondents undertook. On further exploration, the authors found no positive association between levels of neighbourhood green space and the types of physical activity plausibly associated with green space. The positive associations found were in fact due to higher levels of manual work or occupational physical activity among people who happened to be resident in greener areas.

Only a small number of studies have been conducted using a more green space specific physical activity measurement. These tend to be conducted at a regional level, using a smaller number of respondents. Sugiyama et al., for example, explored the association between the perceived greenness of the neighbourhood and walking for recreation; reporting a positive association [21]. Although these studies attempt to capture the salient elements of outdoor physical activity, they were still limited by their inability to conclude whether the reported physical activity actually occurred specifically *in green space.* This highlights the need for research to capture information on the environments in which people are physically active. One way to do this is to use accelerometers or pedometers, coupled to GPS. Such data are able to accurately assess both the quantity and the location of physical activity. There are increasing numbers of such studies available [22-25]. However, the quantity of data produced by such methodologies, and the practical difficulties in running large scale studies, means that they are not well suited to capturing associations between physical activity and environment at the population level.

A further reason for our interest in a population level study of physical activity within green space stemmed from Mitchell and Popham’s observation that socio-economic inequalities in health may be narrower in urban neighbourhoods with relatively more green space, than in those with relatively less [26]. Mitchell and Popham suggested that the equalised opportunities for physical activity which green space offer could be a key mechanism behind this narrowing. It is feasible that those living in a less green area might require transport to, or entry fees for, opportunities to be physically active in a suitable environment. This could produce socio-economic inequalities in physical activity and, in turn, in health. Where green space is plentiful, material resources are no longer a barrier to physical activity, and inequalities in activity, and thus health, are narrowed.

In this study therefore, we were concerned both with assessing the extent to which green space is a venue for physical activity and testing Mitchell and Popham’s hypothesis about its role in narrowing inequalities in physical activity. We drew on data from the 2008 Scottish Health Survey (SHS) which is unusual in combining detailed measures of physical activity quantity, type and location.

Our research questions were (a) is availability of neighbourhood green space positively associated with physical activity and with physical activity specifically in green space, and (b) is the association between socio-economic position and participating in physical activity within green space narrower among people living in greener areas compared to less green areas?

## Methods

### Data

The Scottish Health Survey (SHS) is a large, repeat cross-sectional nationally representative survey designed to provide a comprehensive picture of the health of the Scottish population, including physiological measures and health-related behaviours. The sample was drawn using multistage stratified probability sampling with postcode sectors selected at the first stage and household addresses selected at the second stage. The survey methodology is described in detail elsewhere [27]. Our study included adult respondents aged 16+ years from the 2008 survey.

### Physical activity

We defined three measures of physical activity; overall physical activity, walking, and green physical activity. The first two measures were based on the UK government’s recommendations for levels of physical activity. The SHS asked detailed questions about respondent physical activity levels in the four weeks prior to interview. These included the type, intensity, duration and frequency of activities. The first measure we derived from these items was for overall physical activity and included house work, walking, playing sport and manual work. The measure distinguished those who met the UK recommendations of undertaking at least thirty minutes of moderate or vigorous physical activity on five or more occasions in a week, from those who did not. The second measure was for walking. It identified those who achieved at least thirty minutes of fast or brisk walking on at least five occasions per week. We hypothesised that levels of walking may be particularly sensitive to the quantity of green space available in the neighbourhood.

A subset of SHS respondents (*n* = 2269) were also asked to report the environments they used for physical activity. The options were: local pavement or streets, home/garden, open space/park, country paths, woods/forests, beach/river bank, gym/sports centre, swimming pool, outdoor sports pitch and ‘somewhere else’. The SHS did not capture the duration or intensity of physical activity that were undertaken in each environment, but respondents did report how often they used each environment. Following Mitchell [28], we derived variables that captured the frequency with which respondents used any green environment for physical activity. This method is described in detail elsewhere [28] but, in brief, it summed reported uses of Woods/Forest, Open space/park and/or Non-tarmac paths in the previous four weeks, and converted this to a mean use per week. We were able to define two different measures of use of green space for physical activity, or ‘green physical activity’ as we then labeled it. The first measure defined green physical activity as green spaces “used once a week or more”, the second defined green physical activity as green spaces “used three times a week or more”.

### Neighbourhood green space

We required a measure of availability to green space for each SHS respondent. This was achieved through the specially arranged addition of a green space variable to the publically available SHS dataset. Each respondent in the SHS was matched to an area-level measure describing the quantity of green space available their area of residence. This matching process was undertaken by SHS data managers, to maintain the anonymity of their respondents. The derivation of this green space measurement is described elsewhere [29] and the data are freely available online [30]. In brief, the measure is an estimate of the percentage of green space within each Census Area Statistics (CAS) ward in Scotland. A CAS ward is an administrative unit used to report area statistics in Scotland (mean population 4144, median size 3.5 km^2^). The green space measurement included natural areas (e.g. parks, beaches and agricultural land) but excluded aquatic areas and small water bodies (e.g. lochs, the sea and river corridors) and domestic gardens. Although the variable has been shown to perform well in comparisons with other measures of green space in the UK [31], it performs poorly in rural areas because there is too little variation in the quantity of green space. All our analyses were therefore carried out for residents of urban areas only, as defined by the Scottish Government’s Urban/Rural classification [32].

The green space variable was attached to the SHS respondents via their postcode and the matching was undertaken by the Scottish Government data managers to ensure the anonymity of SHS respondents. For analysis, the green space variable was categorised into four groups (<25, 25- < 50%, 50- < 75%, and 75%+). We undertook analysis to assess the sensitivity of results to different categorisations and in particular to the choice of threshold for the lowest green space category which would form the reference group in out analyses. The substantive findings were unaltered and we therefore present results for this categorisation only.

### Co-variates

We adjusted our models for age, sex and equivalised household income tertile. We adjusted for age and sex because there is good evidence that both physical activity levels and use of green spaces differ by age and sex. Since the relationships between age and physical activity are not linear, we modelled age as a categorical variable (16–24, 25–34, 35–44, 45–54, 55–64, 65–74 and 75+ years). Income, our proxy for socio-economic position, was both a potential confounder in the relationship between physical activity and green space (because wealthier people may both take more exercise, and also be more likely to reside in greener areas), and used as an axis of inequality in physical activity for the part of the study assessing whether inequalities in physical activity were related to neighbourhood green space level. Rates of missingness for income were higher among respondents living in the least green areas. To be certain that excluding these respondents did not influence our results; we performed two further procedures. First, we ran models with an additional category for those who did not report income. Second, we imputed missing income data responses using Stata’s Multiple Imputation by Chained Equations (MICE) procedures. Our substantive findings were not sensitive to the treatment of the income variable and we therefore present results based on excluding all respondents with missing income data.

### Models

To answer research question (a), we explored the association between availability of green space and levels of (i) walking, (ii) overall physical activity and (iii) green physical activity using logistic regression models. We then answered research question (b) by first adding an interaction term to the model which explored whether any association between socio-economic position and physical activity varied by the availability of green space. The resulting coefficients were tested using the Wald test for interaction. The exact nature of any interactions was further unpacked using a sequence of logistic regression models that were stratified by green space.

Multi-level modelling was not possible as the CAS ward of residence was not disclosed to the research team. All analyses were weighted to take account of any differences between the Scottish Population and the SHS sampling strategy. Models were run in Stata (version 11).

## Results

### Sample characteristics

The characteristics of the respondents in our analysis are shown in Table 1 (*n* = 3679).

**Table 1:** Percentage (n) distribution of characteristics of urban Scottish Health Survey respondents (n = 3679) by demographic, socio-economic, green space availability and physical activity variables

**Characteristics of respondents meeting physical activity outcomes**								
	**Characteristics of respondents**		**Meeting physical activity guidelines**		**Meeting walking guidelines**		Participating in green physical activity > 1/wk*	
	**n**	**%**	**n**	**%**	**n**	**%**	**n**	**%**
**Demographic and socio-economic**								
**Characteristics**								
**Sex**								
Male	1621	44.06	669	41.27	311	19.19	185	33.88
Female	2058	55.94	650	31.58	299	14.53	231	33.14
Total	3679	100.00	1319	35.85	610	16.58	416	33.47
**Age**								
16-24	327	8.89	152	46.48	82	25.08	31	28.97
25-34	477	12.97	246	51.57	111	23.27	72	40.68
35-44	669	18.18	306	45.74	136	20.33	99	44.00
45-54	656	17.83	257	39.18	110	16.77	82	39.61
55-64	638	17.34	204	31.97	88	13.79	56	26.79
65-74	521	14.16	119	22.84	65	12.48	56	31.46
75+	391	10.63	35	8.95	18	4.60	20	14.29
Total	3679	100.00	1319	35.85	610	16.58	416	33.47
**Equivalised household income**								
Top tertile (> = £29900)	1274	34.63	555	43.56	249	19.54	171	43.51
2^nd^ tertile (> = £14932 < £29900)	1138	30.93	421	36.99	193	16.96	133	33.93
Bottom tertile (<£14932)	1267	34.44	343	27.07	168	13.26	112	24.45
Total	3679	100.00	1319	35.85	610	16.58	416	33.47
**Ward level green space availability**								
<25%	750	20.39	284	37.87	138	18.40	89	33.09
25- <50%	1356	36.86	497	36.65	225	16.59	136	29.76
50- <75%	481	13.07	166	34.51	78	16.22	50	33.11
75%+	1092	29.68	372	34.07	169	15.48	141	38.52
Total	3679	100.00	1319	35.85	610	16.58	416	33.47

*Measured for a subsample of the Scottish Health Survey respondents (n = 1243).

#### (a) Is availability of neighbourhood green space positively associated with physical activity and with physical activity specifically in green space?

No association at all was observed between neighbourhood green space and meeting the recommended overall physical activity guidelines (data not shown). In un-adjusted models, respondents living in the greenest neighbourhoods were less likely to meet recommended walking guidelines (odds ratio 0.73, 95% CI 0.55-0.95) than those in the least green areas (Table 2a). After adjustment for age, sex and household income, this association was attenuated a little and became non-significant (Table 2a). In both un-adjusted and adjusted models, the odds of meeting the walking guidelines appeared to follow a ‘dose-response’ relationship with neighbourhood green space such that the odds fell further for each gradation of green space. An independent, positive association between household income and meeting the recommended walking guidelines was also observed.

**Table 2:** The association between green space and physical activity outcomes (odd ratios and 95% confidence intervals), obtained from logistic regression models with sequential adjustment for demographic and socio-economic indicators

	**(a) Recommended walking guidelines**		**(b) Green physical activity**	
	**Model 1 (baseline)**	**Model 2 (+ age, gender and income)**	**Model 1 (baseline)**	**Model 2 (+ age, gender and income)**
Green space				
<25%	1.00	1.00	1.00	1.00
25- <50%	0.89 (0.69-1.15)	0.93 (0.71-1.21)	0.81 (0.57-1.15)	0.85 (0.59-1.22)
50- <75%	0.78 (0.55-1.09)	0.80 (0.57-1.13)	0.92 (0.59-1.43)	0.99 (0.62-1.58)
75%+	0.73 (0.55-0.95)*	0.77 (0.59-1.02)	1.11 (0.78-1.59)	1.20 (0.83-1.74)
Gender				
Male		1.00		1.00
Female		0.71 (0.58-0.86)**		0.95 (0.73-1.24)
Age				
16-24		1.00		1.00
25-34		0.77 (0.54-1.10)		1.50 (0.85-2.63)
35-44		0.65 (0.47-0.92)*		1.52 (0.89-2.61)
45-54		0.48 (0.34-0.69)***		1.25 (0.72-2.17)
55-64		0.38 (0.26-0.54)***		0.72 (0.41-1.26)
65-74		0.37 (0.25-0.54)***		1.13 (0.64-1.98)
75+		0.12 (0.07-0.22)***		0.44 (0.22-0.87)*
Income				
Top tertile		1.00		1.00
2^nd^ tertile		0.80 (0.63-1.01)		0.72 (0.53-0.99)**
Bottom tertile		0.77 (0.60-0.99)*		0.48 (0.34-0.67)***

*0.01 _ p < 0.05; **0.001 _ p < 0.01; ***p < 0.001.

No significant association was observed between neighbourhood green space and participation in green physical activity. The odds ratios were inconsistent in direction and gave no indication of a dose–response relationship (Table 2b). Again, an independent, positive association between household income and participation in green physical activity was observed (Table 2b, Model 2). No substantive difference in results was observed when green physical activity was measured as use more than 3 times a week (data not shown).

#### (b) Is the association between socio-economic position and participating in physical activity within green space narrower among people living in greener areas compared to less green areas?

**Figure 1:**
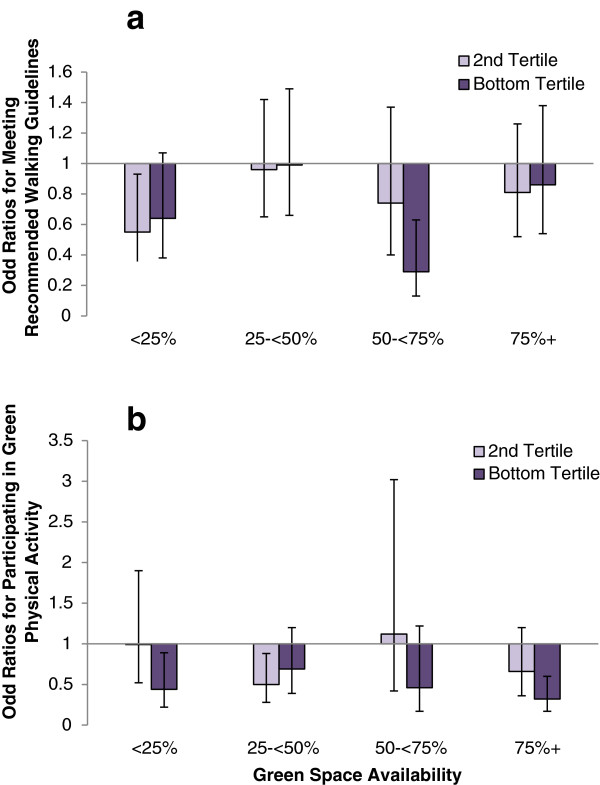
**Does income related inequality in (a) meeting the recommended physical activity guidelines and (b) participating in green physical activity vary by green space availability?** Odds ratios given relative to the reference group (Top Income Tertile, OR = 1.0) and bars indicate 95% confidence intervals.

Figure 1a shows how income-related inequality in meeting the recommended walking guidelines varied by level of neighbourhood green space. The odds of those in the lowest income tertile meeting the recommended walking guidelines compared to those in the highest income tertile were 0.64 (95% CI 0.38-1.09) in the least green areas, and 0.86 (95% CI 0.54-1.38) in the most green areas; neither showing a significant difference. There was a weakly significant interaction such that the income-related gap in meeting the recommended walking guidelines was narrower in the most green areas *(Wald test: x2 = 12.80, P = 0.0463).* However, there was no evidence of a dose response relationship such that the inequality became narrower as green space levels increased; results for the 25- < 50% and 50- < 75% green neighbourhoods were very different. A similar pattern of results was observed for meeting the recommended physical activity guidelines with no indication of any dose response relationship (data not shown).

There was also no significant difference in the association between household income and green physical activity by level of green space availability *(Wald test: x2 = 9.09, P = 0.1687)* (Figure 1b)*.* The odds ratio for participation in green physical activity among those in the bottom income tertile compared with the top income tertile was 0.44 (95% CI 0.22-0.89) in the least green areas and 0.32 (95% CI 0.17-0.60) in the most green areas. Results were similar when green physical activity was defined as three times a week or more (data not shown).

## Discussion

In this Scottish study, the amount of green space in a neighbourhood tended not to be associated with the physical activity levels of urban residents. No significant relationships were found between quantity of green space in a neighbourhood and either meeting recommended walking or physical activity guidelines, or participation in green physical activity. We also found no evidence that income-related inequalities in physical activity, or in green physical activity, were narrower in greener areas of urban Scotland.

This was the first study in the UK, and one of the first in the world, to explore associations between levels of green space in a neighbourhood and physical activity *specifically in green space*, at a population level. The absence of both any relationship between neighbourhood green space and green physical activity, and evidence for narrower inequalities in physical activity in greener neighbourhoods, contradicted our hypothesis that green spaces equalise opportunities for physical activity and that this in turn produces narrower socio-economic health inequalities in greener areas [26]. It is important to understand that the study does not contest the idea that green space promotes physical activity, or that physical activity in green space is healthy; rather it challenges the ideas that (i) any green space in the neighbourhood can act as venue for, and thus encourage physical activity for the residents of that neighbourhood, and (ii) this will occur equally across the socio-economic spectrum.

The lack of association between green space and physical activity in this study is consistent with several recent studies [18,19,33]. In New Zealand for example, neighbourhood access to parks and beaches was not associated with physical activity behaviour [33]. Similarly, no relationship was found between the quantity of green space and meeting recommended physical activity guidelines in the Netherlands [19]. Although Mytton et al. [18] found a positive association between green space and physical activity in England, no association was shown for sub domains of physical activity more likely to take place in green space. Studies focussed specifically on walking behaviour have also shown similar negative results. Giles-Corti et al. [34], for example, found that distance to green space was not associated with achieving the recommended levels of walking.

What might explain our findings? The studies which *have* reported a positive association between green space and physical activity tend to be those which take the aesthetic attributes of the environment into consideration. A recent study by Sugiyama et al. [35] for example, found that presence of an attractive green space in the neighbourhood was positively associated with walking for recreation, but the number of green spaces in the neighbourhood alone was not. Giles-Corti et al. [36] and Sugiyama et al. [37] also found a positive association between the attractiveness and presence of natural features and walking behaviour. These results suggest that the aesthetic attributes of the environment may be key in any relationship between green space and physical activity. Our green space measure, however, contained no information on the quality or attractiveness of green space. We note too that this study is the first to explore the relationship between green space and physical activity specifically in Scotland. The lack of association in our results may therefore, reflect something about the social, cultural or behavioural patterns of physical activity and/or use of green space that is specific to Scotland, differences in the type of green space in its urban areas, or simply Scotland’s ambient climate. Another possible explanation is that physical activity in green space carries greater health benefits for more disadvantaged populations than for the more advantaged. Thus, whilst levels of green physical activity may be lower in less advantaged populations, their impact may be more substantial than for the more advantaged populations.

Our study’s strengths were its ability to examine physical activity specifically in green space, well tested measures of physical activity which relate to clinical recommendations, and a large representative sample matched with an objective measurement of green space exposure, allowing a national level exploration of the association between green space and physical activity in many different urban settings. Our study also had limitations however. First, our three physical activity outcomes were self-reported. Self-reported measures have known disadvantages of incomplete recall and over-estimation of physical activity. However, the threat to validity would stem from an association between recall bias and green space availability. This seems unlikely. Second, our green space measurement was somewhat crude. We had to assume that respondents residing in neighbourhoods with the same amount of green space actually had equal access to that green space and that the green spaces were all useable for physical activity. Our measurement of green space, for example, included agricultural land; areas which can be regarded as unsuitable for physical activity. However, as the study was conducted in urban Scotland only, having included sufficient agricultural land to influence our results remains highly unlikely. At the time of writing no national dataset describing the different classifications of green spaces was available in Scotland. However, a new data set which would be able to distinguish spaces suitable for physical activity has very recently become available and could be a useful resource for future work [38]. Third, green physical activity reported by respondents may not necessarily have occurred within their neighbourhood green space. Individuals may spend a large proportion of their day outside their neighbourhood. Identifying exactly where individuals were physically active, as opposed to what type of environment, was not possible with the data available. Fourth, our data were cross-sectional and residual confounding cannot be discounted. Fifth, there may have been some important confounding variables that we were unable to account for in our models. We had no information about green space quality, neighbourhood density, concerns about safety and crime or the aesthetic attributes of the environment, for example.

The main implication of our study is that in Scotland physical activity may not be the main mechanism behind associations between green space and population health which have been observed in the UK and elsewhere[2,3,7,29], or behind the narrowing of socio-economic inequalities in health in greener neighbourhoods observed in England by Mitchell and Popham [26]. When we survey other candidate mechanisms, the direct effect of perceiving natural environments on physiological and psychological health would appear the more plausible explanation. Those effects have been demonstrated in laboratory and field experiments [39,40] and it is reasonable to assume that they operate at a population level too.

## Conclusion

This was one of the first population level studies to explore the association between amount of green space in urban neighbourhoods and the population’s use of green space for physical activity. Our findings suggest that increasing the quantity of green space in a neighbourhood in the assumption it will a venue for physical activity, could be wrong. A deeper understanding of the mechanisms involved would help policy makers and planners create a more health-promoting natural environment.

## Competing interests

The authors declare that they have no competing interests.

## Authors’ contributions

RM, JP and KO conceived the study. KO conducted the statistical analysis and drafted the manuscript. All authors participated in the interpretation of the data and approved the final manuscript.
